# A novel technique for pectoral enhancement performed during excisional gynaecomastia procedures

**DOI:** 10.1016/j.jpra.2026.07.037

**Published:** 2026-07-31

**Authors:** Neala Glynn, Rachel Currie, Nicholas Hodgins

**Affiliations:** Department of Plastic Surgery, Ulster Hospital, Belfast, Northern Ireland, Ireland

**Keywords:** Gynaecomastia, Pectoral augmentation, Body contouring

## Abstract

The presence of gynaecomastia can result in significant psychological distress. Surgical intervention should aim to remove glandular tissue whilst ensuring an aesthetically pleasing result. We present a case of excisional gynaecomastia correction with simultaneous enhancement of the pectoral contour with a modified dermal flap. This method utilises otherwise redundant autologous tissue without an additional donor site. We report an aesthetically pleasing result and discuss other modalities of pectoral enhancement currently described in the literature.

## Introduction

Gynaecomastia, which results in glandular tissue deposition in the male breast can result in significant psychological distress.^1^ Gynaecomastia can be described utilising the Simon criteria which grades severity from one to three based on the degree of breast enlargement and presence of skin redundancy.^2^^,^^3^

In the preoperative setting, it is imperative to be mindful of the functional and psychological factors which prompted a surgical consult. Surgical intervention to address gynaecomastia should aim to remove excess glandular tissue and ensure a pleasing aesthetic result.

Here we present a case of gynaecomastia excision with autologous pectoral enhancement and review alternative techniques of gynaecomastia correction and pectoral enhancement currently described in the literature.

## Aims

**Fig. 1:**
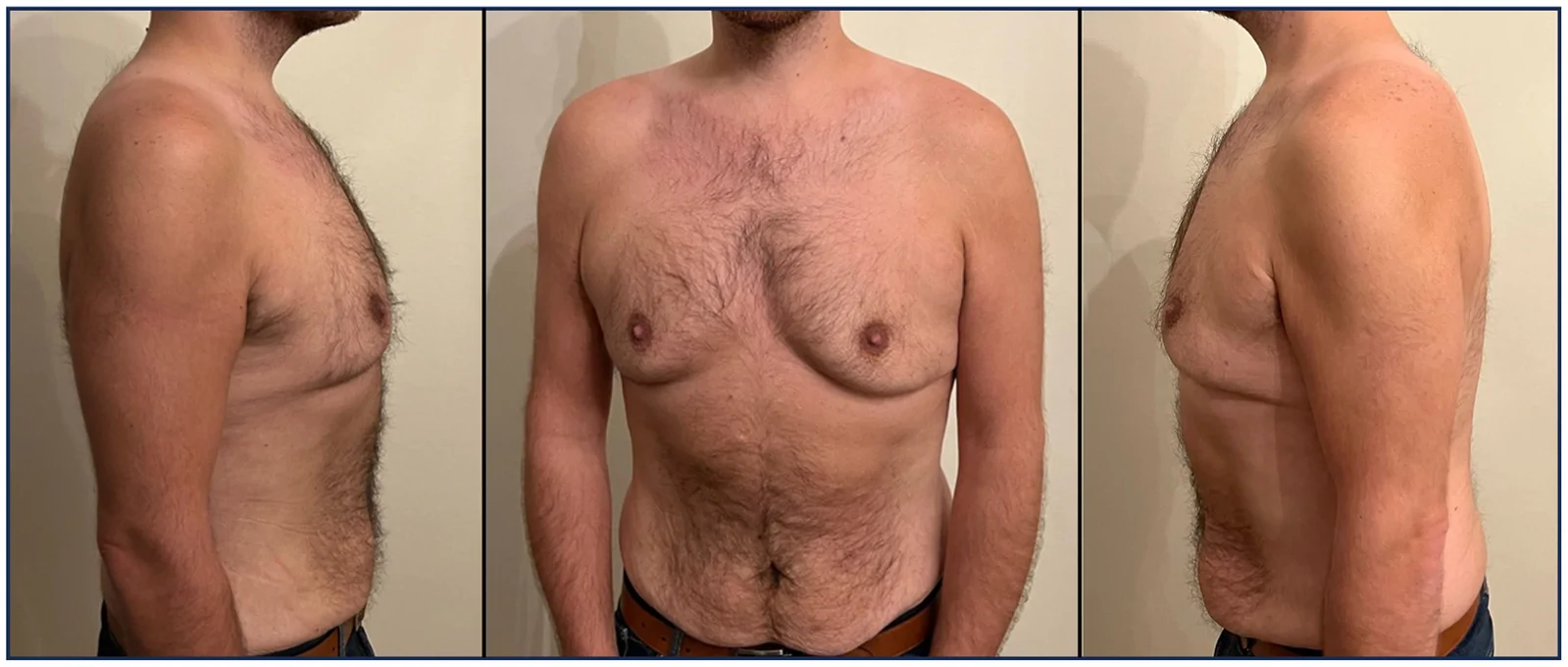
Pre-operative photographs.

We present the case of a 35-year-old male with idiopathic bilateral gynaecomastia and a normal hormonal profile. Excess skin on his chest was exacerbated following 11 stone weight loss. On examination, bilateral grade 3 gynaecomastia was noted with significant skin redundancy in the vertical vector and greater volume on the right (Fig. 1). The patient was boarded for a bilateral nipple preserving mastectomy.

## Methods

**Fig. 2:**
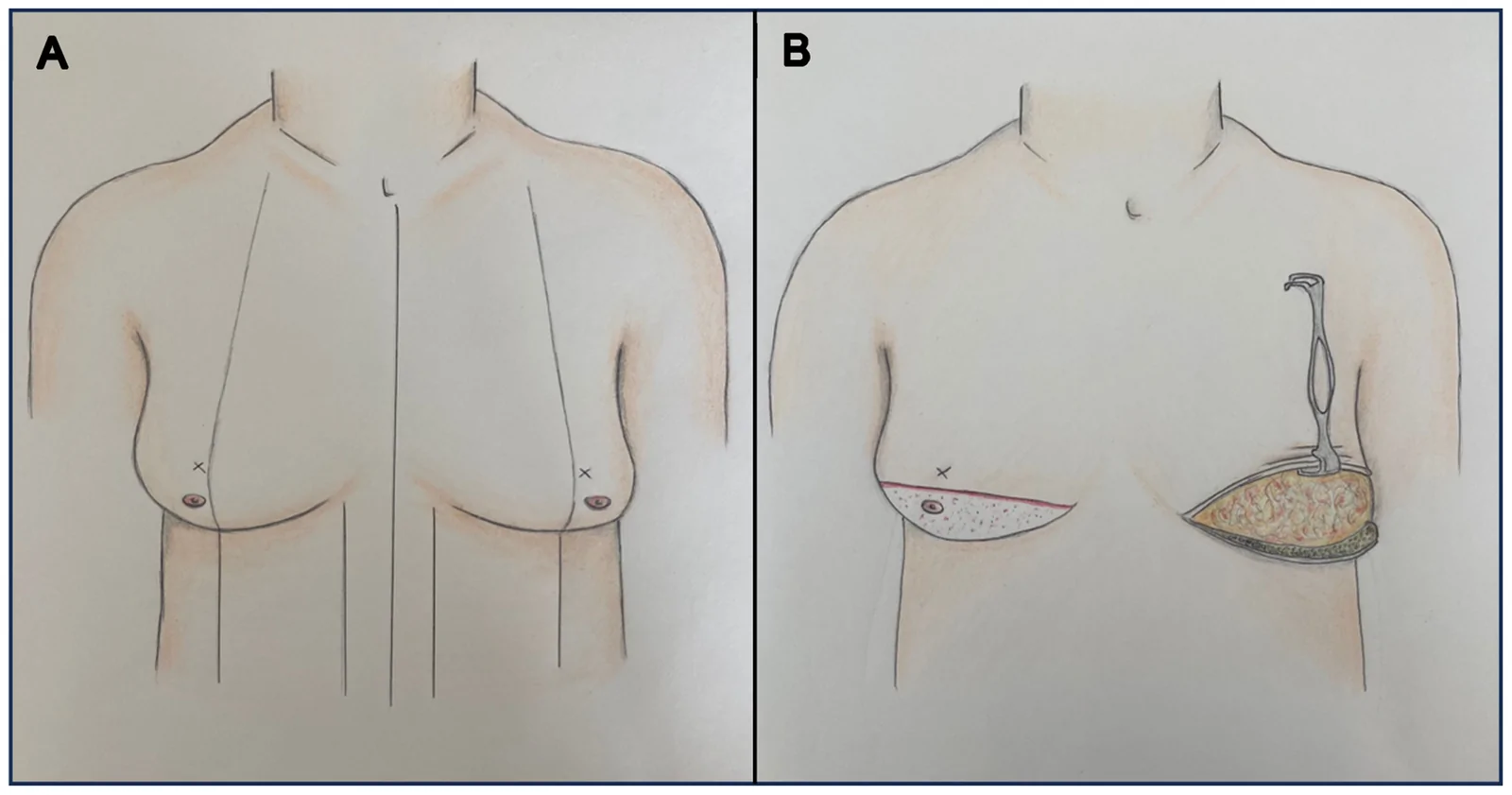
A: Preoperative markings. B: De-epithelialisation to create dermal flap (illustrated on right breast) and incision of the flap superiorly to perform mastectomy (illustrated on left breast).

The patient was marked standing. The midline, infra-mammary fold and breast base were marked. The new nipple areolar complex (NAC) position was marked (Fig. 2A).

An ellipse was marked on the breasts which was subsequently de-epithelialised, preserving the NAC. The superior perimeter of the ellipse from the 9 o’clock to 3 o’clock position was incised to access the breast tissue. Skin flaps were raised superiorly and inferiorly in the mastectomy plane. In total, 185g of tissue was excised from the right breast and 150g from the left (Fig. 2B).

**Fig. 3:**
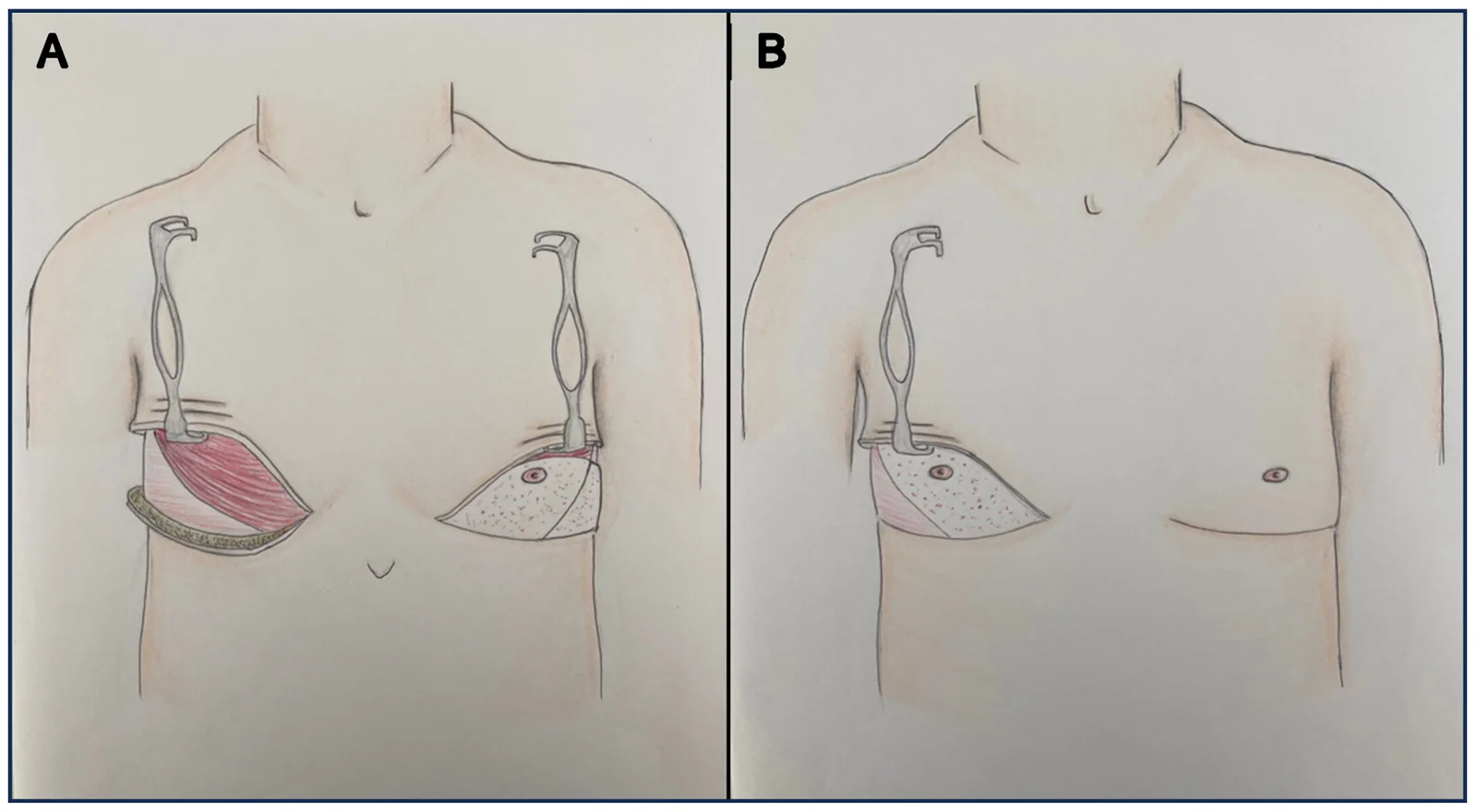
A: Appearance following mastectomy (illustrated on right breast) and redraping of dermal flap with NAC on inferior pedicle (illustrated on left breast). B: Excision of redundant dermal flap laterally (illustrated on right breast) and redraping of skin with aperture for the NAC (illustrated on left breast).

De-epithelialised tissue lying below the inferolateral border of the pectoralis major was excised. This ensured that the remaining dermal flap mirrored the contour of the underlying pectoralis major muscle (Fig. 3A). The inferiorly based dermal flap was fixed to the fascia of pectoralis major, thus augmenting soft tissue cover. The superior skin flap was laid down and an aperture for the NAC created (Fig. 3B).

The incision was extended onto the lateral chest wall to excise lateral standing cones. A ¼ inch drain, skin glue, incisional negative pressure dressing and compression garment were utilised.

**Fig. 4:**
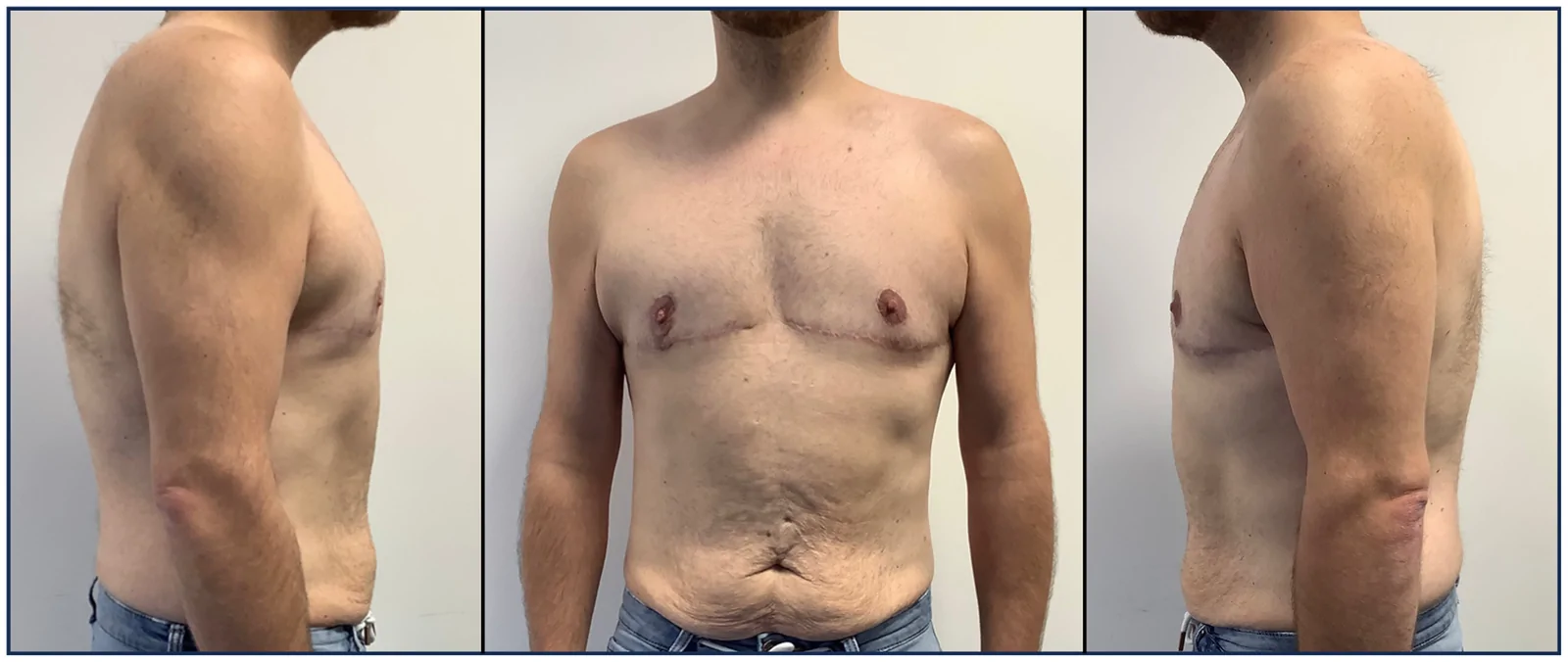
Post operative photographs.

A small left sided haematoma and small right sided seroma were present within the early post operative phase, these were managed conservatively. At 10 months post-op the resulting chest contour was good with slight loss of right nipple projection. The patient was very satisfied with the appearance of his chest (Fig. 4).

## Discussion

Standard techniques of gynaecomastia excision include minimally invasive techniques, mastectomy or breast amputation with a free nipple graft.^2^ Mastectomy with skin excision is commonly utilised in severe cases including Simon grades II-III. Skin excision can also be utilised in instances where a reduction in the size of the NAC is desired.^2^

The degree of soft tissue redundancy prompted an open technique in our case. When removing skin in combination with glandular tissue, care must be taken to preserve the vascular supply to the nipple. We preserved the NAC on an inferior pedicle similar to the gynaecomastia inferior pedicle reduction technique (GIRT) described initially by Kornstein et al.^4^ Our simple modification of excision of the dermal flap inferior to the pectoralis major muscle resulted in additional enhancement of the pectoralis major contour, which is previously undescribed in this setting.

A technique described by Wyrick et al. coined the ‘double doughnut’ allows for access through a circumareolar incision with excision of a ring of skin and preservation of the NAC on a superior pedicle.^5^ A similar technique described by Ibraheim et al. use this incision with adjunctive liposuction.^6^ The circumareolar incision may prove aesthetically acceptable to patients albeit there is a risk of circumareolar scar stretch. The open technique in our case allowed for the dermal pedicle for the NAC to be fashioned to augment the projection of the pectoralis major which is not possible with a circumareolar approach.

Standard techniques of pectoral augmentation include implants and lipofilling. The use of implants has reported complications similar to those seen in breast augmentation including haematoma, seroma, capsular contracture and implant migration or exposure.^7^ The use of autologous tissue removes these risks, however, the volume of enhancement achieved with our technique is not comparable to an implant.

Body contouring techniques include modalities such as laser, cryolipolysis, radiofrequency, liposuction and pectoral etching.^8^ Pectoral etching is a technique whereby the outline of the muscle is enhanced with fat contouring. Whilst this may be an acceptable means of aesthetic enhancement in the normal male chest this technique would not address the glandular tissue or ptosis associated with gynaecomastia. Liposuction may be utilised to address fatty gynaecomastia but is less effective in glandular breasts and does not address skin redundancy.

Use of autologous tissue for pectoralis enhancement at the time of gynaecomastia surgery is described by Pilanci et al.^9^ In their series, patients underwent gynaecomastia excision followed by intramuscular pectoral fat injections with abdominal fat. The authors proposed that this enhanced the pectoral outline and reduced the uneven contour sometimes experienced by patients. Two early instances of fat necrosis were encountered which settled with massage.

Similar techniques utilising fat grafting following gynaecomastia excision are described by Hoyos et al.^10^ In their series, they combined gland removal with lipofilling in a multilayer fashion to achieve a contoured pectoral profile. The lipofilling technique requires an abdominal donor site and the risks associated with liposuction must be considered.

## Conclusion

This case demonstrates a novel technique for pectoral enhancement at the time of gynaecomastia excision. The use of autologous tissue negates the need for an implant or secondary donor sites. We demonstrate an aesthetically pleasing result postoperatively and recommend this technique be considered as an adjunct in open gynaecomastia excision.

## Declarations

The authors have no conflicts of interest to declare.

No financial support or ethical approval was required for preparation of this article.

Written informed consent to publish the medical images was obtained from the case study subject.
